# Recognize Yourself—Innate Sensing of Non-LTR Retrotransposons

**DOI:** 10.3390/v13010094

**Published:** 2021-01-12

**Authors:** Justine Lagisquet, Kilian Zuber, Thomas Gramberg

**Affiliations:** Institute of Clinical and Molecular Virology, Friedrich-Alexander University Erlangen-Nürnberg, 91054 Erlangen, Germany; justine.lagisquet@uk-erlangen.de (J.L.); Kilian.Zuber@fau.de (K.Z.)

**Keywords:** LINE-1, Alu, autoimmune disease, inflammation, nucleic acid sensing, PRR

## Abstract

Although mobile genetic elements, or transposons, have played an important role in genome evolution, excess activity of mobile elements can have detrimental consequences. Already, the enhanced expression of transposons-derived nucleic acids can trigger autoimmune reactions that may result in severe autoinflammatory disorders. Thus, cells contain several layers of protective measures to restrict transposons and to sense the enhanced activity of these “intragenomic pathogens”. This review focuses on our current understanding of immunogenic patterns derived from the most active elements in humans, the retrotransposons long interspersed element (LINE)-1 and Alu. We describe the role of known pattern recognition receptors in nucleic acid sensing of LINE-1 and Alu and the possible consequences for autoimmune diseases.

## 1. Introduction

In the years and decades following Barbara McClintock’s seminal work in the 1950s on mosaicism in maize [1], it became clear that mobile genetic elements, or transposons, are a major part of all eukaryotic genomes. Evolutionary speaking, replicating genetic elements are very successful, and in the case of the human genome, repetitive, transposon-derived sequences make up almost 50% of its size [2]. Depending on the type of transposon, these elements mobilize from one locus within the genome to another through either a DNA- or a RNA-intermediate [3]. DNA transposons, grouped into class II elements, replicate via a “cut-and-paste” mechanism, with DNA “jumping” into a new integration site. In contrast, class I elements, or retrotransposons, replicate via an RNA intermediate, in a “copy and paste”-like fashion by reverse transcribing their RNA directly into the host DNA at a new genomic location. Previously disregarded as just being junk DNA, it is now evident that transposable elements played, and still play, an important role in shaping structure and function of our genome [4,5]. However, depending on the site of integration, retrotransposition events can also destabilize genome integrity and cause disease [6,7,8]. Thus, cells have evolved multiple countermeasures to hold retroelements at bay and developed mechanisms to recognize retrotransposition as potential threat. Most transposons found in the human genome became inactivated over the course of evolution. The only actively transposing elements known in humans today belong to the group of non-LTR retrotransposons. Thus, this review summarizes the current knowledge on innate sensing of the most abundant active elements within the human genome, long interspersed element (LINE)-1, and Alu elements.

## 2. Mobile Genetic Elements in Humans

**Figure 1:**
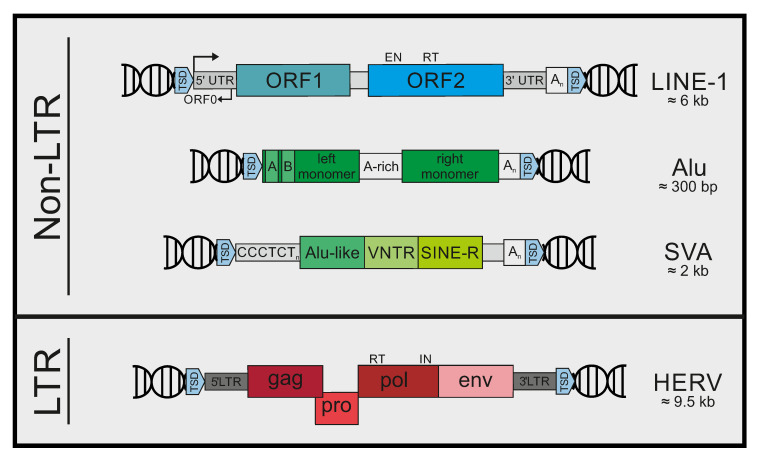
Types of retrotransposons in humans. Non-long terminal repeat (LTR) retroelements include long interspersed element (LINE)-1 elements, Alu elements, and short-interspersed element (SINE)-variable tandem repeats (VNTR)-Alu (SVA) elements. Full-length LINE-1 elements are approximately 6 kb in length and encode two open-reading frames (ORF1 and ORF2) in sense orientation, flanked by untranslated regions (UTR). LINE-1 also contains an antisense open reading frame (ORF0) within the 5′UTR, in which the internal promoter is located. The 3′UTR includes a poly(A) signal. The 300 bp Alu elements are composed of a left and right monomer separated by an adenosine-rich sequence (A-rich). The left monomer contains an internal RNA polymerase III promoter (A and B boxes). The structure of SVA elements starts with a (CCCTCT)_n_ hexamer repeat region, which is followed by an Alu-like domain, a variable tandem repeats (VNTR) region, a human endogenous retrovirus (HERV)-K-derived SINE-R domain, and a poly(A) signal. LTR retrotransposons mainly include HERVs. HERV sequences consist of two LTRs flanking four open reading frames, coding for structural proteins (*gag*), protease (*pro*), polymerase and reverse transcriptase (*pol*), and the envelope protein (*env*), which is mostly dysfunctional. Abbreviations: TSD, target site duplication; EN, endonuclease; RT, reverse transcriptase; gag, group-specific antigen; IN, integrase.

Within the genome of humans and other mammals, sequences derived from class I retrotransposons make up the great majority of repetitive elements [4]. They can be categorized into two main groups: LTR retroelements, characterized by the presence of long terminal repeats (LTR) at both ends, and non-LTR retroelements, lacking the aforementioned repeats (Figure 1). The group of LTR-retroelements comprises the different families of human endogenous retroviruses (HERVs) [9,10], and even older types of elements, such as the mammalian apparent LTR-retroelements (MaLRs) [11]. Altogether, sequences derived from LTR-retroelements make up approximately 8% of the human genome [2]. HERVs are relics of ancient retroviruses that found their way into the germline and eventually became fixed in the human population [9]. Thus, intact HERV genomes strongly resemble exogenous retroviruses and are composed of LTRs flanking the retroviral open reading frames (ORFs) *gag, pro*, *pol*, and *env.* In humans, active retrotransposition of LTR-elements has been extinguished within the last few million years. However, in certain cell types and tissues, HERV RNA and protein expression can still be found, and some HERV-derived proteins have even been domesticated and fulfill important functions in humans today [12]. In addition, an aberrant expression of HERV transcripts has been observed in various inflammatory diseases and tumor samples. For example, a role of HERV transcripts in autoinflammatory diseases such as multiple sclerosis (HERV-W) or in cancer development (HERV-K) has been suggested (reviewed in [13,14]). In general, HERVs have been highly mutated over the course of millions of years of vertical transmission, and most of the time only solitary LTRs can be detected within the human genome. Today, none of the known HERVs are considered replication-competent, although members of the HERV-K (HML-2) group have a relative intact proviral sequence [15,16].

The group of non-LTR retrotransposons is formed by long interspersed elements (LINEs) and short-interspersed elements (SINEs). LINE-derived sequences account for approximately 21% of the human genome, and LINE-1 elements, the principal component of this family, are the only transposons left today that are able to replicate autonomously [2,4]. LINE-1 contains two main ORFs, encoding ORF1p, a structural protein with known RNA-binding activity, and the enzyme ORF2p, which harbors the LINE-1 endonuclease and reverse transcriptase activities [4,7,17]. The 5’UTR (untranslated region) of LINE-1 contains the RNA polymerase II-dependent promotor, and a poly(A) site is located within the 3′UTR. In addition, LINE-1 contains a small antisense ORF (ORF0), located within the 5′UTR, which seems to be responsible for the enhancement of LINE-1 mobility [18]. Upon RNA polymerase II-mediated transcription and nuclear export of the mRNA, translation occurs. Newly synthesized ORF1p and especially ORF2p preferentially bind to “their own” LINE-1 mRNA in *cis* to form ribonucleoprotein complexes (RNPs) [19,20]. These complexes are then relocated to the nucleus, where LINE-1 initiates reverse transcription and integration into a new genomic location by a process called target-primed reverse transcription (TPRT) [21]. Interestingly, LINE-1 integration into the human genome is not random. The endonuclease ORF2p of LINE-1 cleaves host DNA at the defined target sequence 3′-AATTTT-5′. Thus, LINE-1 preferentially inserts into nucleosome-free DNA due to its higher A-T content sequences [22]. In addition, DNA replication seems to affect LINE-1 insertion and cleavage of the lagging strand within DNA replication forks is common [23]. In contrast to LINE-1 elements, SINEs are non-autonomous and rely on LINE-1 activity to complete retrotransposition. They represent about 13% of the human genome and contain the family of Alu elements, which are the most frequent SINE elements in humans, as well as SINE-variable tandem repeats (VNTR)-Alu (SVA) elements and others [4]. Alu elements are non-protein coding sequences of 300 base pairs derived from the 7SL RNA. They have a dimeric structure with two monomers separated by an adenosine-rich linker sequence. An internal RNA polymerase III-dependent promoter drives RNA transcription. Upon nuclear export, Alu transcripts bind to LINE-1 ORF2p and hijack its endonuclease and reverse transcriptase activities to complete its own retrotransposition [24,25]. Although retrotransposition of both LINE1 and Alu elements contributes to genome diversity and flexibility, *de novo* insertions can also have devastating effects, depending on the site of integration [26].

## 3. Controlling Mobile Elements

Host species have developed various countermeasures to control retrotransposons on different levels. The first layer of protection is epigenetic silencing. Here, transcriptional silencing of the retrotransposon promotor is mediated by DNA methylation and adding repressive histone modifications through various protein complexes, such as human silencing hub (HUSH) complex [27,28], KAP1/TRIM28 [29,30,31], or polycomb repressor complex 2 (PCR2) [32]. Furthermore, posttranscriptional regulation by microRNAs (miRNA) or piwi-interacting RNAs (piRNA) has been shown to control retrotransposition. In germline cells of higher organisms, piRNAs target transcripts of mobile elements by antisense base pairing and initiate their degradation via piwi protein complexes [33]. In somatic cells, miRNAs, such as miR128, have been shown to limit LINE-1 activity by directly degrading its RNA or by limiting the expression of crucial cofactors [33,34,35]. In addition, cells have developed various restriction factors that inhibit retroelements posttranslationally. Comprehensively summarized by John Goodier in a recent review [36], many host factors originally identified as antiviral restriction factors also interfere with various steps of retrotransposition. Here, we only briefly highlight some of these factors. APOBEC3 proteins are cytidine deaminases known to hypermutate retroviral nucleic acids [37]. In humans, the APOBEC3 family consists of seven members (A3A-D, A3F-H), all of which have been shown to inhibit both LTR and non-LTR elements, at least in cell culture models, with A3A and A3B being the most effective [38,39]. In contrast to exogenous retroviruses, however, the mechanism of restriction seems to be different for retrotransposons and, for most members of the family, to be deaminase independent [40]. SAMHD1 is a dNTP hydrolase known to regulate intracellular dNTP levels in a cell cycle-dependent manner and to restrict the replication of many viruses in non-dividing cells by limiting the number of available dNTPs needed to replicate viral genomes (recently summarized in [41]). The mechanism how SAMHD1 restricts retrotransposons seems also to differ from its activity against exogenous viruses, with different potential models of restriction being proposed [42,43,44,45]. The three prime repair exonuclease 1 (TREX1) has been shown to prevent retroviral cDNA accumulation in the cytoplasm of infected cells [46]. Upon HIV-1 infection, TREX1 removes excess reverse transcription products and prevents sensing of HIV cDNA by cytoplasmic DNA sensors, thereby boosting HIV-1 infectivity. Interestingly, Stetson et al. demonstrated that the lack of TREX1 in mice led to a strong accumulation of endogenous retroelement nucleic acids in inflamed heart cells, and that the overexpression of TREX1 inhibited the LINE-1 reporter construct activity in vitro [47]. Another inhibitory factor, the RNA helicase MOV10, interacts with LINE-1 RNA in RNPs, thereby interfering with LINE-1 reverse transcription and integration into the genome [48]. The zinc-finger antiviral protein ZAP has been described to specifically bind to high CpG-containing RNA sequences [49]. Binding of ZAP to CpG-rich HIV-1 RNA has been shown to inhibit infectivity by activating a cellular nuclease that degrades viral RNA. Interestingly, ZAP also represses non-LTR retroelements in a process involving RNA and ORF1p interaction, most likely also resulting in LINE-1 RNA degradation [50,51].

## 4. Non-LTR Retrotransposons in Inflammatory Diseases

Even with this flurry of protective measures at hand, *de novo* insertions occur in germ line and somatic cells. Although a certain degree of retrotransposition might add an additional layer of flexibility to genome evolution, development, as well as to gene regulation, excess activity of mobile elements can have detrimental consequences. Novel insertions can disrupt open reading frames of genes, modify the activity of enhancers and promoters, change splicing patterns, provoke gene duplication, or mediate recombination, causing different diseases depending on the sites affected. Insertional mutagenesis resulting in monogenetic disorders or cancer is the most straight forward example how retrotransposon activity can cause disease and over 120 such cases have been described to date [7]. Most famously, in 1988 Kazazian and colleagues identified a LINE-1 insertion in the coagulation factor VIII gene in the genome of a haemophilia A patient, demonstrating for the first time that retrotransposons are still active in humans today [52]. 

In addition to insertional mutagenesis, the enhanced expression of retroelements has also been linked to various disorders. Especially in inflammatory diseases, non-LTR retrotransposon activity has been suggested to play an important role in pathogenesis. Here, the accumulation and sensing of excess nucleic acids derived from retrotransposons is seen as potential trigger for autoimmune responses, similar to exogenous virus infection (Table 1). The role of retrotransposons has been extensively studied in case of the rare neuroinflammatory disorder Aicardi–Goutières syndrome (AGS). AGS presents itself in newborns, closely resembling a congenital viral infection, and is characterized by enhanced interferon (IFN) level in the cerebrospinal fluid, a strongly reduced life expectancy, and severe physical and mental impairments [53]. Loss-of-function mutations in genes that play a role in nucleic acid metabolism have been associated with the disease, such as *TREX1* (AGS 1), the subunits of *RNaseH2* (AGS 2-4), *SAMHD1* (AGS 5), *ADAR* (AGS 6), and the RNA sensor *IFIH1/MDA5* (AGS 7) [54]. 

Systemic Lupus Erythematosus (SLE) is an autoinflammatory disorder in which a systemic autoimmune reaction causes widespread inflammation and tissue damage. Among other potential causes, it has also been hypothesized that enhanced LINE-1 activity might trigger the initial autoimmune reactions, which then culminate in the inflammatory phenotype of SLE. Interestingly, in some cases of SLE, TREX1 deficiency has been linked to the disease, suggesting a similar mechanism of triggering autoimmune responses in SLE and AGS [55,56]. 

Fanconi anemia (FA) is a very rare genetic disorder that is characterized by progressive bone marrow failure, cancer, and features associated with accelerated aging. On a molecular level, a defective DNA damage response is thought to lead to elevated levels of inflammatory mediators associated with the disease [70,71]. In addition, Brégnard and colleagues found that LINE-1 single-stranded DNA (ssDNA) accumulates in the cytoplasm of cells from FA patients, providing an additional explanation for the autoimmune response in FA [64]. Although not a disease in the classical sense, age-related enhanced inflammation is well described. Using human cell culture and murine animal models, two independent studies recently suggested that LINE-1-derived nucleic acids contribute to the enhanced inflammatory phenotype in aged cells [66,67]. Geographic atrophy (GA) is an advanced form of age-related macular degeneration (AMD) and is characterized by the decay of retinal pigmented epithelium (RPE) cells, resulting in the loss of vision. An accumulation of Alu elements has been reported in patients suffering from GA, which might contribute to the pathogenesis by activating the NLR family pyrin domain-containing 3 (NLRP3) inflammasome [68,72]. Sjögren’s syndrome is an autoimmune condition that can manifest itself at any age and affects women 10 times as often as men. It is a relatively benign autoimmune disorder; however, it often appears as a complication of other autoimmune diseases, such as rheumatoid arthritis or SLE [73]. The trigger of the disease is still unknown but symptoms in patients include dry eyes and mouth. Multiple sclerosis (MS) is an autoinflammatory disease affecting the central nervous system [74]. During MS, the myelin sheath of axons is attacked by the host’s immune system, causing significant physical disability in over 30% of patients aged 20–25 years. Typically, in MS symptomatic episodes occur, which might be months or even years apart and can affect different anatomic locations. The exact cause for MS is still unclear. Interestingly, among other potential candidates, chronic virus infections (various herpesviruses) as well as an enhanced activity of endogenous retroviruses, such as HERV-W, have been suggested to play a role in triggering the disease [14].

## 5. Sensing of Mobile Genetic Elements

A deregulated expression of endogenous retrotransposons has been reported for many autoinflammatory disorders and interferonopathies and has been suggested to play a critical role in their pathogenesis [53]. In most cases, we only begin to understand how transposable elements trigger innate sensing and inflammatory pathways and how sensing might contribute to the various phenotypes of the respected diseases. Due to the nature of transposable elements, nucleic acids of some form are the most obvious patterns recognized by innate receptors. Here, we summarize the progress of the field on innate sensing of endogenous retrotransposons and the cellular receptors involved. We focus mainly on the immunogenic patterns of LINE-1 and Alu elements, the only actively transposing elements in humans. The sensing of LTR retroelements and HERVs has been excellently addressed in several recent reviews [6,75,76,77,78]. 

Of note, although we are focusing on endogenous retroelements, the accumulation of nucleic acids from these elements is not the only molecular pattern discussed to trigger inflammatory diseases such as AGS or SLE. Alternatively, uptake of extracellular DNA or enhanced level of cytoplasmic DNA resulting from aberrant DNA repair or replication processes have been correlated with autoimmune reactions by several groups [79,80]. 

## 6. Pattern Recognition Receptors

In general, various complementary systems exist to detect “non-self” nucleic acids. Here, we briefly introduce those pattern recognition receptors (PRRs) that have been implicated in retroelement sensing. Toll-like receptors (TLR) are extracellular or endosomal PRRs that scavenge the cellular environment for conserved pathogen-associated molecular patterns (PAMPs) [81]. Members of the TLR family are predominantly active in specialized immune cells. Expression patterns of the different TLRs are cell type-specific and tailored to the needs of the respective cell type. The nucleic acids sensing TLRs 3 and 7 are most prominently expressed in endosomes of phagocytic immune cells, such as monocytes, macrophages, and dendritic cells (DCs), and have been implicated in LINE-1 and Alu sensing. Within the endosomal compartment, nucleic acids that are usually covered within viruses become available for sensing upon digestion. TLR3 detects double-stranded RNA (dsRNA) molecules, while the TLR7 has been shown to be activated by ssRNA. 

In addition, various intracellular PRRs are set up to detect non-self nucleic acids in the cytoplasm. The well-described group of RIG-I-like receptors (RLRs) sense the presence of unusual dsRNA structures in the cytoplasm and include the retinoic acid-inducible gene I (RIG-I/DDX58), the laboratory of genetics and physiology 2 (LGP2/DHX58), and the melanoma differentiation-associated gene 5 (MDA5/IFIH1) (reviewed in [82]). RLRs belong to the DExD/H family of RNA helicases and share a similar structural build and sequence. In addition to their central RNA helicase domain, which mediates RNA binding, RIG-I and MDA5 contain two amino-terminal CARD domains (2CARD) that are essential for initiating immune signaling. In contrast, LGP2 is lacking both CARD domains and is therefore thought to play a regulatory role of the bona fide receptors RIG-I and MDA5 [83]. Upon binding of viral or host target dsRNA, RIG-I and MDA5 multimerize and interact with the adapter molecule mitochondrial antiviral signaling (MAVS) to initiate downstream signaling, which culminates in type I IFN and proinflammatory cytokine expression. Although both RLRs bind dsRNA and share a high structural similarity, RIG-I and MDA5 recognize an almost complementary range of viral and cellular targets. In general, RIG-I favors rather short, blunt-ended, and uncapped dsRNA molecules containing a 5′ tri- or di-phosphate moiety. Well-known physiological ligands include viral ssRNAs containing stem loop or panhandle structures, present for example during replication of Sendai virus, influenza A virus, or dengue virus. On the other hand, MDA5 recognizes long dsRNA molecules (1 kb and longer), on which it forms filaments upon binding to induce signaling. Physiological targets of MDA5 include dsRNA intermediates occurring during replication of ssRNA viruses such as picornaviruses [84,85].

Intracellular DNA sensing has been rather obscure for a long time, and a variety of different candidates for sensing DNA have been proposed over the years. While some of them might only serve as DNA sensors under certain conditions or in specialized cell types, two factors have been generally accepted as bona fide DNA sensors, IFNγ-inducible protein 16 (IFI16) and cyclic GMP-AMP synthase (cGAS) [86]. IFI16 belongs to the family of pyrin and HIN domain-containing (PYHIN) proteins and can be found in the nucleus or the cytoplasm, depending on the cell type. IFI16 has been shown to initiate innate signaling pathways upon sensing of HIV-1 in macrophages and T cells [87,88] or herpesviral DNA in the nuclei of infected cells [89,90,91]. The role of IFI16 in sensing endogenous retroelements is still ill described. However, IFI16 has been shown to sequester the transcription factor Sp1, leading to inhibition of LINE-1 promotor activity and retrotransposition in vitro [92]. The extensively studied PRR cGAS senses DNA of cellular and viral origin, including reverse transcribed cDNA of HIV-1 [93,94]. Upon binding of cytoplasmic dsDNA, cGAS synthesizes the second messenger cGAMP, which binds and activates the adapter molecule stimulator of IFN genes (STING) [95]. Interestingly, IFI16 also fuels into the STING signaling pathway [87]. Upon activation through either DNA receptor, STING initiates downstream signaling cascades, resulting in the enhanced transcription of proinflammatory genes and type I IFN via IFN regulatory factor 3 (IRF3) and NF-κB activation. In addition, cGAS can also be found in the nucleus, where it has been shown to sense HIV DNA with the help of the cellular cofactor NONO [96]. However, the mechanism of activation of nuclear cGAS sensing is still unclear, since it has been shown to be efficiently inactivated by chromatin structures to prevent autoimmune reactions targeting chromosomal DNA [97]. 

Protein kinase R (PKR) is an IFN-inducible dsRNA-dependent kinase localized in the cytoplasm that recognizes viral RNA and directly suppresses viral replication [98]. PKR is a monomeric protein that undergoes autophosphorylation and dimerization upon binding of dsRNA [99]. PKR sensing requires dsRNA of at least 30 bp in length or unmodified 5′ tri-phosphate groups at short 5′ and 3′ overhangs. Subsequently, the activated kinase phosphorylates several downstream target proteins, including the translational initiation factor eIF2. Phosphorylated eIF2 is no longer able to mediate initiator tRNA shuttling to ribosomes, resulting in a general shut down of mRNA translation and therefore to a block of viral and host protein synthesis. 

NLRP3 belongs to the family of NOD-like receptors (NLR) and is expressed mainly in macrophages, where it functions as an activator of the NLRP3 inflammasome. NLRP3 is a tripartite protein that contains an amino-terminal pyrin domain (PYD), a central nucleotide-binding and oligomerization domain (NOD), and a C-terminal leucine-rich repeat (LRR). It has been shown to mediate caspase-1 activation, resulting in the secretion of proinflammatory cytokines IL-1β/IL-18 in response to microbial infection and cellular damage (reviewed in [100]). Although the exact mechanism of activation is still not clear, NLRP3 inflammasomes seem to be triggered by diverse stimuli, such as mitochondrial dysfunction, ionic flux, or reactive oxygen species.

## 7. Immunogenic Patterns in LINE-1

**Figure 2:**
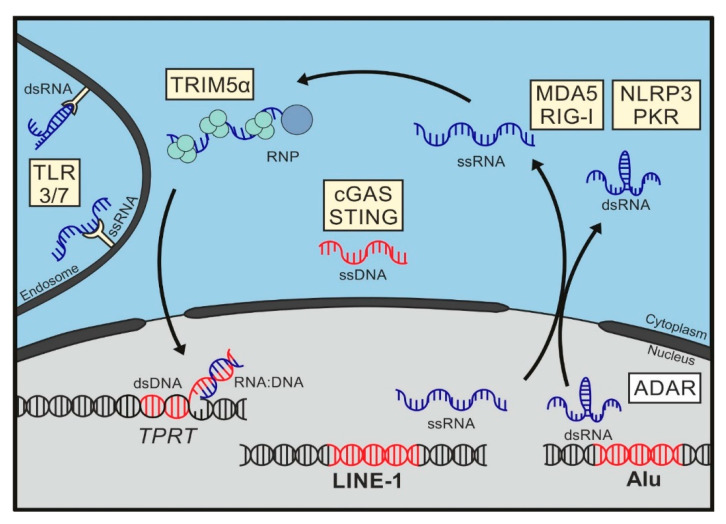
Sensing of LINE1 and Alu elements. Here, we give an overview of the different nucleic acids of LINE-1 and Alu elements and how they can be sensed by pattern recognition receptors (PRRs, yellow boxes) during retrotransposition. In the nucleus, LINE-1 and Alu sequences are transcribed by RNA polymerase II (LINE-1/Alu) or III (Alu), resulting in single-stranded RNA molecules (ssRNA). Upon export to the cytoplasm, ssRNAs of both elements have been shown to activate double-stranded RNA (dsRNA) sensors, such as melanoma differentiation-associated gene 5 (MDA5) or retinoic acid-inducible gene I (RIG-I) under certain conditions. After translation, LINE-1 ribonucleoprotein complexes (RNPs) are formed and are composed of *de novo* synthesized ORF1p and ORF2p and the RNA molecule they have been translated from. Accumulation of excess RNPs is sensed by human tripartite motif-containing 5α (TRIM5α) in the cytoplasm. RNPs are imported into the nucleus, where the process of target-primed reverse transcription (TPRT) takes place to allow integration of LINE-1 at a new genomic location. During this process, genomic DNA is cleaved by ORF2p, liberating a 3′OH group, which is used to initiate reverse transcription. This might result in the formation of immunogenic RNA/DNA hybrid structures. LINE-1 single-stranded DNA (ssDNA) has been shown to accumulate in the cytoplasm, triggering an activation of the cyclic GMP-AMP synthase (cGAS)-STING pathway. It has been suggested that LINE1 ssDNAs are products of aberrant reverse transcription, which are exported to the cytoplasm. Pol II transcripts containing inverted Alu elements embedded in 3′UTRs of other genes can form double-stranded RNA molecule (dsRNA), which is usually highly modified by ADAR1 and mainly retained in the nucleus. In case of ADAR1 deficiency, Alu dsRNA structures can access the cytoplasm and are sensed by different PRRs, such as protein kinase R (PKR), MDA5, RIG-I, but also Toll-like receptor 3 (TLR3). In addition, Alu transcripts have been shown to trigger NLRP3 inflammasome activation and to bind to TLR7 under certain conditions, which might contribute to the pathogenesis of autoimmune disorders, such as systemic lupus erythematosus.

LINE-1 elements are the only autonomously replicating transposons still active within the human genome. During LINE-1 retrotransposition, newly transcribed RNA is incorporated into ribonucleoprotein complexes (RNPs), transferred into the nucleus, and reverse transcribed into LINE-1 cDNA at a new genomic location. Thus, LINE-1 RNA, RNPs, ssDNA, and dsDNA, as well as nicked genomic DNA generated during this process are potential immunogenic nucleic acids that could be targeted by different innate receptors. Indeed, different species of LINE-1 nucleic acids have been described as triggers of inflammatory responses. The different findings might not be as contradictory as they seem at first glance, as cellular sensors might recognize the activation of LINE-1 retrotransposition at different steps or in a cell type-specific manner (Figure 2). 

### 7.1. LINE-1 DNA

The most compelling evidence for a LINE-1-associated pattern inducing inflammatory responses has been collected for LINE-1 DNA. Already in 2008, Stetson and colleagues identified LINE-1 DNA as a potential trigger of autoinflammation in TREX1 knockout (KO) mice [47]. Loss-of-function mutations in TREX1 are associated with Aicardi–Goutières Syndrome (AGS), a disease that clinically mimics congenital viral infection [101]. In mice, TREX1 deficiency results in a pronounced autoimmune phenotype, and KO mice die at a very young age due to circulatory failure instigated by a pronounced myocarditis [102]. To identify the nature of nucleic acids causing the autoimmune response in mice, Stetson and colleagues isolated and cloned cytoplasmic DNA from homogenized hearts of wild type (WT) and TREX1 KO mice and found a strongly enhanced accumulation of DNA corresponding to LTR retroelements, LINE elements, and SINE elements in the KO samples [47]. Since no overlapping sequences of the same element were identified, Stetson et al. suggested ssDNA to be the trigger of the autoimmune response. In addition, overexpression of TREX1 in in vitro reporter assays blocked retrotransposition of LINE-1 and the LTR retroelement IAP, further corroborating the inhibitory effect of TREX1 on retrotransposition. Due to the enhanced LINE-1 DNA accumulation in the absence of TREX1 in vitro, reverse-transcribed DNA from retroelements are thought to induce, or at least to contribute to, sterile inflammation in TREX1-deficient mice in vivo. However, treating TREX1 KO mice with the HIV reverse transcriptase (RT) inhibitor AZT did not ameliorate autoimmunity [47]. In contrast, an independent study by Beck-Engeser and colleagues found that feeding TREX1-deficient mice an FDA-approved cocktail of different RT inhibitors strongly attenuated myocarditis and significantly prolonged the survival of the mice [103], suggesting that reverse transcription of retroelements contribute to autoimmunity in TREX1 AGS. Achleitner et al. tried to reproduce the results in TREX1 KO mice in a very detailed analysis but did not detect a dampened autoinflammation or prolonged survival of the mice upon treatment with the same inhibitors [104]. The reason for the discrepancies between the studies is not clear. One caveat might be an unspecific immunosuppressive effect of the RT inhibitors, which might attenuate inflammation independently of retroelement activity. Moreover, far more different LTR and non-LTR elements are active and replicate in mice compared to the human genome. Thus, it is conceivable that RT inhibitors designed to block HIV do not inhibit all murine endogenous retroelements, further complicating the analysis in this animal model. 

In another study analyzing autoimmunity in the absence of functional TREX1, Thomas and colleagues generated pluripotent stem cells from AGS patients to analyze the consequences of TREX1 deficiency in human neuronal cells [57]. The authors found enhanced cytotoxicity in neurons and elevated type I IFN release from astrocytes lacking TREX1, resembling the clinical phenotype of AGS in patients. In TREX1-deficient cells, such as neuronal progenitor cells, the authors observed highly elevated levels of ssDNA accumulating in the cytoplasm, a major part of which were of LINE-1 origin. The accumulation was sensitive to RT inhibitors, suggesting that the excess ssDNA in these cells is generated by reverse transcription, most likely by enhanced LINE-1 activity. The authors found a stronger phosphorylation of the transcription factor IRF3 in TREX1-deficient cells, which was reversible by RT inhibitors. Since IRF3 is phosphorylated upon STING signaling, Thomas et al. suggested that cGAS/STING-mediated sensing of LINE-1 DNA triggers the autoimmune reaction in the absence of TREX1 [57]. The authors speculated that stem-loop formation or any other secondary structures within LINE1 ssDNA activate cGAS. Indeed, the cGAS/STING pathway has been shown to be activated by LINE1 DNA in the context of other autoimmune diseases as well, such as Fanconi anemia.

Fanconi anemia (FA) is a genetic disorder caused by mutations in one of 17 known genes involved in DNA repair and characterized by autoinflammation and enhanced cytokine production. In 2016, Brégnard et al. analyzed, on a molecular level, SLX4 deficiency, which is associated with FA in patients, in order to identify the trigger for the chronic inflammation [64]. SLX4 plays a pivotal role in assembling host DNA repair complexes but has also been shown to initiate pathogen DNA degradation to reduce the immune response towards these molecules [105,106]. In FA patient-derived cell lines, Brégnard et al. observed increased levels of LINE-1 ssDNA in the cytoplasm. The accumulation of cytosolic ssDNA in FA cells was reversible by adding RT inhibitors, suggesting that the immunogenic DNA in FA is reverse transcribed. Interestingly, the authors found that autoinflammation in the absence of SLX4 is strongly reduced upon KO of cGAS or STING, suggesting that both proteins are crucial for LINE-1 sensing in FA.

In addition to genetic disorders, recent studies uncovered an essential role of enhanced LINE-1 activity in aging. De Cecco and colleagues identified LINE-1 elements as drivers of IFN-mediated, age-associated inflammation [66]. Using a human fibroblast model, the authors found that LINE-1 transcription is strongly activated during cellular senescence. They were able to confirm these findings in mice and found enhanced levels of LINE-1 RNA and protein in murine cells from older animals. The authors found that cytoplasmic cDNA is triggering the immune response and that the inflammatory reactions can be reversed by RT inhibitors. Moreover, the authors discovered that the type I IFN response in these cells is abrogated upon cGAS or STING knockdown, further corroborating the importance of the DNA sensing pathway in detecting enhanced LINE-1 activation. Interestingly, among other genes, the expression of TREX1 was strongly reduced in senescent cells, suggesting that the loss of TREX1-mediated reduction of LINE-1 nucleic acids might contribute to age-associated inflammation.

In another age-related study, Simon and colleagues analyzed SIRT6 KO mice, which exhibit a severely shortened lifespan and growth retardation [67]. The lysine deacetylase and ribosylase SIRT6 is central to heterochromatin formation and gene regulation. Among other functions, SIRT6 has been shown to ribosylate KAP1/TRIM28, which in turn downregulates LINE-1 promotor activity by heterochromatin formation [29]. Similar to previous work, the authors found strongly elevated levels of cytoplasmic LINE-1 DNA in SIRT6 KO mice, as well as in tissues and cells from aged WT mice, a phenotype that was sensitive to RT inhibitor treatment [67]. They also found that increased LINE-1 activity and cytoplasmic LINE-1 DNA level correlated with upregulated type I IFN activity in these mice. Targeting the DNA sensor cGAS by shRNA, reduced type I IFN level again. In addition, the authors cross-linked and precipitated cGAS from murine fibroblasts and found a significantly increased number of LINE-1 DNA molecules bound to cGAS from SIRT6 KO cells compared to cGAS from WT cells. Together, these results suggest that also during aging, LINE-1 DNA might trigger autoinflammatory responses by activating the cGAS-STING pathway.

Following up on the idea that the sensing of endogenous reverse-transcribed DNA might contribute to AGS, Rice et al. initiated a first human trial testing the effect of FDA-approved RT inhibitors in AGS patients [107]. The authors administered a combination of three RT inhibitors to eight patients over a period of 12 months. In these patients, RT inhibitor treatment led to a decrease in inflammatory markers, such as IFN-α level in serum and plasma, downregulation of IFN signaling, as well as a reduction of IFN-stimulated gene (ISG) expression. Thus, these promising first results in AGS patients further support the idea that reverse transcription of endogenous retroelements might contribute to autoinflammatory disorders, such as AGS, in humans.

Together, there is plenty of evidence now *suggesting* that excess reverse-transcribed LINE-1 DNA triggers innate immune reactions via the cGAS/STING pathway. Most studies, however, identified LINE-1 ssDNA accumulation as the most likely PAMP, and it is still unclear as to how cGAS, a known dsDNA sensor, is stimulated by LINE1 ssDNA. The most likely explanation would be that cGAS senses secondary structures, such as stem loop formations, in LINE-1 ssDNA or partially double-stranded LINE-1 cDNA, possibly resulting from aberrant reverse transcription and integration processes. In an alternative explanation, the retroelement-derived nucleic acid triggering the cGAS-mediated immune response is dsDNA and the presence of immunostimulatory dsDNA might only be overshadowed by excess ssDNA accumulation in the in vitro models discussed above. 

### 7.2. LINE RNA

In addition to DNA, some studies suggest that RNA molecules might be the immunostimulatory entities in LINE-1. Zhao and colleagues reported that endogenous LINE-1, in the absence of inhibitory microRNA, triggers IFN-β production in human cell lines independently of LINE1 DNA synthesis or LINE-1 retrotransposition, making LINE-1 RNA the most likely immunostimulatory nucleic acid [58]. Fittingly, they found the immune stimulation by LINE-1 to be independent of cGAS and STING but highly dependent on RNA sensing pathways, such as MDA5 and RIG-I. Knockdown of MDA5 as well as RIG-I in human cell lines reduced IFN-β expression in the face of activated LINE-1. In addition, overexpression of the known AGS-associated proteins ADAR1, TREX1, RNaseH2, and SAMHD1 seemed to suppress the LINE1-triggered immune activation. Thus, the authors suggest that LINE-1 RNA sensing by MDA5 and RIG-I contributes to innate immune activation in autoimmune diseases such as AGS [58]. However, the molecular details as to how LINE-1 RNA activates both sensors are still unclear. In principle, LINE-1 RNA should be very similar to other cellular mRNAs, containing 5′ cap structure and a poly(A)-tail. Thus, the immunostimulatory nature of LINE-1 RNA needs to be analyzed in the future. Supporting a potential role of LINE-1 RNA in autoimmune diseases, Mavragani and colleagues found enhanced LINE-1 mRNA levels in renal biopsies from multiple sclerosis patients and salivary gland biopsies from Sjögren’s syndrome patients [62]. 

### 7.3. LINE-1 RNA/DNA Hybrids

Another immunogenic form of LINE-1 that might play a role in human AGS has been identified by Lim and colleagues [59]. Using state-of-the-art sequencing approaches, the authors analyzed the genomes of primary fibroblasts from AGS patients with mutations in TREX1, RnaseH2, and SAMHD1. In these genomes, the authors identified a loss in overall DNA methylation together with a strong accumulation of RNA/DNA hybrid structures compared. Interestingly, analyzing these structures revealed a significant enrichment of sequences corresponding to LINE-1 and LTR retrotransposons. However, it is still unclear how these RNA/DNA hybrid structures are formed and how these structures trigger innate immune responses. The ribonuclease complex RNaseH2 is important for preventing ribonucleotide incorporation and degradation of RNA/DNA hybrids within the host genome. However, in contrast to Lim et al., Benitez-Guijarro and colleagues found that, unlike other AGS factors, RNaseH2 does not inhibit LINE-1 retrotransposition but rather promotes the integration of new LINE-1 copies, at least in vitro, possibly by degrading RNA present in duplex structures during integration [108]. This finding suggests that nucleic acids of a different origin and not LINE-1-derived nucleic acids trigger AGS in the absence of RNaseH2. Thus, it is very much conceivable, that nucleic acids of different nature (ssDNA vs. RNA) and origin (retroelements, aberrant DNA replication and repair) might trigger different immune pathways, resulting in a very similar phenotype to AGS.

### 7.4. LINE-1 RNPs 

Recently, we identified a novel retroelement-specific trigger of innate immune signaling. Our study demonstrated that the intrinsic restriction factor human tripartite motif-containing 5α (TRIM5α) senses and inhibits LINE-1 retrotransposition [109]. Previously, TRIM5 proteins have been shown to restrict exogenous retroviruses in a species-specific manner (reviewed in [110]). Mechanistically, TRIM5α binds and multimerizes around retroviral cores, leading to premature uncoating of the retroviral core and thereby to a block of infection. Interestingly, this scaffold formation of TRIM5α around viral cores has been shown to trigger innate immune signaling pathways, resulting in NF-κB and AP-1 activation [111]. Volkmann et al. found that human TRIM5α also efficiently represses LINE-1 retrotransposition. TRIM5α interacts with LINE-1 ribonucleoprotein complexes in the cytoplasm, which is essential for restriction. In line with its postulated role as pattern recognition receptor, we showed that TRIM5α induces innate immune signaling upon interaction with LINE-1 ribonucleoprotein complexes. The signaling events activate the transcription factors AP-1 and NF-κB, leading to the downregulation of LINE-1 promoter activity, most likely indirectly by activating additional inhibitory factors. Together, we identified LINE-1 as target of TRIM5α, which restricts and senses LINE-1 RNPs in the cytoplasm, leading to inactivation of its promotor via a negative feedback loop, thereby protecting the genome from excess LINE-1 activity [109].

## 8. Immunogenic Patterns in Alu Elements

Alu elements are widespread across the human genome but preferentially concentrate within gene-rich regions [2]. Although the Alu promoter only enables RNA polymerase III driven transcription, Alu elements are often transcribed by RNA polymerase II as well, when their RNA is embedded in a larger transcript, for example in 3′UTRs of neighboring genes. Upon Pol II-mediated transcription, adjacent Alu elements can form long intramolecular dsRNA duplexes, composed of two inverted Alu sequences, commonly referred to as inverted-repeat Alu elements (IR-Alu) [112]. In general, IR-Alu-containing RNAs are usually retained in the nucleus [113]; however, binding to the RNA interacting protein staufen-1 (STAU1) has been shown to mediate the export of embedded IR-Alu RNAs into the cytoplasm. At the same time, STAU1-binding prevents sensing of Alu duplexes by the dsRNA-activated protein kinase PKR, which otherwise would mediate general translational repression [114]. IR-Alu elements are also major targets of adenosine deaminases acting on RNA (ADAR) proteins [115,116], which recognize dsRNA structures and catalyze adenosine-to-inosine RNA deamination resulting in the formation of imperfect Alu duplexes. 

Interestingly, inactivating mutations of ADAR1 are also associated with the autoimmune disorder AGS. Mice deficient for ADAR1 die during embryonic development and show widespread signs of apoptosis and strongly enhanced type I IFN expression [117]. The cause of the autoimmune response, however, was not understood until the discovery of RLR sensing pathways. In ADAR KO mice, the massive autoinflammation can be rescued by introducing an additional KO of the cytosolic dsRNA sensor MDA5 or its adapter molecule MAVS [118,119]. Since IR-Alu duplexes are known targets of ADAR1, a role for murine SINE elements in the activation of the MDA5-dependent autoimmune response in the absence of ADAR1 has been suggested [118,120]. However, since mice lack human Alu elements, Chung and colleagues generated human ADAR1 KO cell lines as well as human embryonic stem cells devoid of ADAR1 to study its role in autoimmunity and IFN regulation [60]. Using RNA sequencing, the authors were able to characterize the editome of ADAR1, and identified Pol II-transcribed Alu elements, located mainly in introns or 3′UTRs, as the main target of ADAR1. These findings suggest a model in which ADAR1 editing prevents spontaneous autoimmune reactions otherwise triggered by the sensing of endogenous IR-Alu complexes via the cytoplasmic sensors MDA5 and PKR. Importantly, gain-of-function mutants of MDA5 have been reported to lead to aberrant activation of the dsRNA sensor causing autoimmune disorders, such as SLE [121,122,123]. A recent study by Ahmad and colleagues corroborated the role of Alu sensing by MDA5 and ADAR1 deficiency in the autoimmune disorder AGS [61]. Analyzing the activity and RNA substrates of AGS-related MDA5 mutants in the presence or absence of ADAR1, the authors identified IR-Alu elements as a major ligand of MDA5 and found that AGS mutants of MDA5 bind these duplexes even more efficiently, even if these elements were edited by ADAR1. On the contrary, WT MDA5 only recognized IR-Alu duplexes in the absence ADAR1 editing, when the duplexes are more prompt to form regular structures due to better base pairing. Together, these studies highlight the loss of tolerance of the cytosolic sensor MDA5 to endogenous IR-Alu elements in AGS and at the same time fortify the role of unmodified Alu duplexes in the absence of ADAR1 as trigger of AGS [61]. However, it is currently not clear how RNA duplexes derived from relatively short Alu elements (300 nt) activate MDA5, a dsRNA sensor that is known to most efficiently detect dsRNA molecules larger than 1000 nt. The explanation might lie in the nature of Pol-II-transcribed IR-Alu duplex structures. Ahmad et al. did not observe an enrichment of all Alu sequences upon MDA5 pulldown but predominantly of those in inverted repeat configuration [61]. Thus, it is tempting to speculate that duplex formation of two inverted Alu elements separated by several 100 nt on a single Pol II transcript might generate an RNA structure large enough to be sensed by MDA5. Such panhandle-like structures in negative strand RNA viruses have been shown to activate RIG-I sensing. It is therefore conceivable that similar structures, maybe with a higher dsRNA content, might also trigger MDA5-dependent immune activation. 

Sensing of Alu elements might also play a role in the pathogenesis of multiple sclerosis (MS). Tossberg and colleagues were able to describe a general decrease in adenosine-to-inosine (A-to-I) editing in leukocytes from MS patients [65]. Using an RNA sequencing approach, they specifically found higher A-to-I editing in Alu elements in samples of healthy donors compared to MS patients. Unedited dsRNA Alu complexes were strongly upregulated in leukocytes from MS patients, correlating with an enhanced type I IFN and NF-κB transcriptional response. In contrast to the AGS and SLE models described above, however, the authors found that unedited Alu elements were not sensed by MDA5 but rather by RIG-I and TLR3 [65]. Of note, sensing of Alu elements by RIG-I has also been observed in certain breast cancers by an independent group [124], further corroborating the findings of Tossberg et al. Why unedited Alu elements are sensed by different pathways in different disease models is currently unclear. Solving the puzzle might help to clarify why some autoimmune disorders triggered by similar nucleic acids differ substantially in their phenotype.

Patients with geographic atrophy (GA) suffer from a decay of the retinal pigmented epithelium (RPE), which might lead to vision loss. Kaneko and colleagues report a deficiency for DICER1, which is involved in host miRNA processing, in RPE cells from GA patients [72]. By immunoprecipitating dsRNA and subsequent sequencing, the authors identified Pol III-transcribed Alu elements to be enriched in RPE cells from patients. The accumulation of Alu transcripts in the absence of DICER correlated with increased cytotoxicity, which could be reversed by targeting the Alu elements with antisense oligonucleotides [72]. The mechanism involved in Alu RNA-mediated toxicity has been further studied by Tarallo and colleagues, who found that the elements were not recognized by MDA5, PKR, or TLRs but rather activate the NLRP3 inflammasome [68]. Interestingly, Pol III Alu transcripts were found to be upregulated in GA, which did not contain IR-Alu duplex structures but single elements. Thus, it is unclear how Alu transcripts activate the NLRP3 inflammasome, possibly by forming intramolecular immunogenic patterns or by initiating indirect danger signals. Kerur and colleagues further analyzed NLRP3 activation by Alu RNA and described a non-canonical NLRP3 inflammasome pathway to be involved in sensing of Alu transcripts, including activation of human caspase-4 and cGAS-mediated signaling [69]. The authors suggest that cGAS recognizes mitochondrial DNA (mtDNA) released in the cytosol due to the Alu RNA triggered opening of the mitochondrial permeability transition pore (mPTP). Together, these findings suggest that an indirect activation of cGAS and NLRP3 in response to Alu elements might be involved in the pathogenesis of geographic atrophy.

Systemic Lupus Erythematosus (SLE) is an autoimmune disease that is characterized by the presence of autoantibodies mainly targeting nucleic acids and nucleic acid-binding proteins (reviewed in [125]). Autoantibodies against the RNA-binding protein Ro60 can be found in SLE as well as Sjögren’s syndrome patients [126,127]; however, the role of Ro60 in the pathogenesis of these diseases remains unclear. Using nucleotide crosslinking and immunoprecipitation (iCLIP) assays, followed by high-throughput sequencing, Hung and colleagues identified an association of Ro60 with Alu RNA [63]. The authors analyzed Ro60-deficient cell lines and found upregulated ISG transcripts as well as enhanced expression of Alu transcripts, suggesting a potential immunogenic role for Alu elements in the absence of Ro60. Upon transfection of peripheral blood mononuclear cells (PBMCs) with Alu RNA, the authors found the TLR7 pathway to be activated, resulting in the secretion of inflammatory cytokines. In line with this finding, the authors observed higher levels of Alu transcripts within blood cells from SLE patients compare to healthy controls. Altogether, this suggests a model, in which the aberrant sensing of Alu transcripts in the absence of Ro60, for example by TLR7, contributes to endogenous immune activation in autoinflammatory diseases such as Sjögren’s syndrome or SLE [63].

## 9. Outlook 

Due to scientific and technological advances, more and more evidence has accumulated over the last couple of years, showing that the uncontrolled activity of endogenous mobile elements has even more detrimental consequences than previously anticipated. In addition to insertional mutagenesis, the enhanced activity of retrotransposons seems to play a major role in triggering autoimmune responses and to contribute to pathogenesis of many autoinflammatory disorders. Although in many cases it is difficult to establish causality between autoimmunity and enhanced retrotransposon activity, the research summarized here suggests that, in addition to aberrant genome repair or replication events, retrotransposon-derived nucleic acids can contribute to autoimmune activation in many cases.

PRRs are known sensors of viral nucleic acids and activate innate immune mechanisms to eliminate the threat to the host. In addition to exogenous viruses, many of these receptors have been shown to sense the activity of endogenous mobile elements, such as LINE-1 and Alu, by recognizing nucleic acids of these elements. In case of viruses, PRRs are triggered by unusual forms of nucleic acids, such as uncapped dsRNA, or by nucleic acids in the wrong location, such as cytoplasmic DNA. In addition, expression levels above a certain threshold are required for a nucleic acid to become immunogenic. However, in case of mobile genetic elements, it is not always clear how cells overcome the difficulty of discriminating between nucleic acids from retroelements and “regular” nucleic acids. Another unsolved but more philosophical question is whether cells actually “want“ to sense the replication of endogenous elements to protect the integrity of their genome or whether the autoimmune response is rather a misguided antiviral response that is accidentally triggered by enhanced replication of mobile genetic elements. In any case, identifying immunogenic patterns in mobile elements and the mechanism of sensing by cellular PRRs is important to understand the molecular basis of autoimmune disorders. Deciphering the role of retroelements and the pathways leading to autoimmune activation might eventually allow for therapeutic intervention in patients suffering from such diseases. A first promising trial using licensed HIV-1 RT inhibitors has already been completed. Identifying molecular patterns and immune sensing pathways involved in a specific autoimmune disorder might enable the use of additional, more specific inhibitors targeting particular PRRs or their subsequent adapter molecules in the future.

## Figures and Tables

**Table 1:** Innate sensing of molecular patterns in non-LTR retrotransposons and its potential role in autoinflammatory phenotypes.

Disease	Element	Nucleic Acid	Sensor	Reference
**Aicardi-Goutières syndrome (AGS)**				
Type 1 (TREX1)	LINE-1	ssDNA	cGAS/STING	[57]
		RNA	MDA5/RIG-I	[58]
		RNA:DNA	unknown	[59]
Type 2-4 (RNaseH2)	LINE-1	RNA	MDA5/RIG-I	[58]
		RNA:DNA	unknown	[59]
Type 5 (SAMHD1)	LINE-1	RNA	MDA5/RIG-I	[58]
		RNA:DNA	unknown	[59]
Type 6 (ADAR)	LINE-1	RNA	MDA5/RIG-I	[58]
	Alu	IR-Alu	MDA5/PKR	[60]
Type 7 (MDA5)	Alu	IR-Alu	MDA5	[61]
**Systemic Lupus Erythematosus (SLE)**	LINE-1	RNA	unknown	[62]
	Alu	RNA	TLR7	[63]
**Sjögren’s syndrome**	LINE-1	RNA	unknown	[62]
**Fanconi Anemia**	LINE-1	ssDNA	cGAS/STING	[64]
**Multiple sclerosis**	Alu	dsRNA	RIG-I/TLR3	[65]
**Aging**	LINE-1	ssDNA	cGAS/STING	[66,67]
**Age Macular Degeneration-Geographic Atrophy (GA)**	Alu	RNA	NLRP3/cGAS	[68,69]

## Data Availability

Not applicable.
